# Relationship between cytomegalovirus antibody levels and cognitive performance is dependent on age and genetic risk

**DOI:** 10.1186/s12883-025-04279-1

**Published:** 2025-07-01

**Authors:** Michael Vacher, Silvia Lee, Patricia Price, Phuongnhi Ha, Shelley Waters, Simon M. Laws

**Affiliations:** 1 CSIRO Health and Biosecurity, Australian E-Health Research Centre, Floreat, Australia; 2 Centre for Precision Health, Edith Cowan University, Joondalup, Australia; 3 School of Medicine, Curtin Medical Research Institute, Curtin University, Bentley, Australia; 4 Department of Microbiology, Pathwest Laboratory Medicine, Murdoch, Australia; 5 Collaborative Genomics and Translation Group, School of Medical and Health Sciences, Edith Cowan University, Joondalup, Australia

**Keywords:** Cytomegalovirus, *APOE* genotype, *TNF* genotypes, Alzheimer’s disease, Cognition, Age-associated cognitive performance

## Abstract

Human cytomegalovirus (CMV) is endemic worldwide. It is often acquired in childhood and persists throughout adult life. CMV is linked with several diseases of aging, but associations with cognitive performance are not consistent. Here we address whether this may reflect a dependence on putative genetic determinants, Apolipoprotein E (*APOE*) ε4 and Tumour Necrosis Factor (*TNF*), and/or the age of the subjects tested. CMV-reactive antibodies were quantitated in 419 individuals aged 71.7 [53.2—89.1] years, drawn from the Australian Imaging, Biomarker & Lifestyle (AIBL) study. Cognitive composite scores, covering five domains, a global score of cognitive performance, brain amyloid-β (Aβ) burden and demographic data were available. *APOE* and *TNF*-308 (rs1800629) genotypes were extracted from genome-wide array data. Bivariate and multivariate analyses were applied. CMV antibody levels were negatively correlated with Aβ burden and correlated directly with cognition in participants carrying the minor allele of *TNF*-308, notably in those lacking the *APOE* ε4 allele. Cognitive performance exhibited a decrease with age, but CMV antibody levels were maintained. Regression analyses revealed significant interactions between *TNF-308* genotype and CMV antibody levels in the overall cohort and in participants younger than 71.1 years (median split). No such interaction was observed in those older than 71.1 years. Overall, CMV antibodies may play a protective role in carriers of the minor allele of *TNF*-308. This was significant in younger individuals and could be eclipsed by advanced age or carriage of the *APOE* ε4. The interactions described may explain disagreements in the literature regarding the effects of CMV and *TNF*-308.

## Introduction

Human cytomegalovirus (CMV) is a herpesvirus that causes persistent asymptomatic infections in healthy individuals and is now linked with many diseases of aging [33]. Associations with cardiovascular disease have been confirmed by meta-analyses [12, 22] and mechanistic studies link the replication of CMV in the blood vessels with the initiation of atherosclerotic plaques [18]. Accordingly, higher levels of CMV-reactive antibodies predicted a loss of vascular elasticity in renal transplant recipients but not healthy adults, presumably because transplant recipients have a higher burden of CMV [15]. Associations with age-associated cognitive impairment are less clear, even though CMV was isolated from the brains of many patients with vascular dementia [20]. One meta-analysis described weak positive and negative associations between CMV antibodies or CMV DNA and cognitive impairment [31]. CMV seropositivity was linked to a faster rate of cognitive decline in statistical models that controlled for age, sex, education, race, vascular risk factors, vascular diseases and apolipoprotein E (*APOE*) ε4 genotype [3]. However, a large cohort study found a non-significant positive association between seropositivity and cognitive loss which became significant in those with poor education [27]. Similarly, CMV seropositivity at recruitment (mean age 61 years) did not predict the development of Alzheimer’s disease (AD) over a 10-year follow-up [21].

We and others have investigated associations with genetic variation in genes of the central major histocompatibility complex (MHC) with a focus on *TNF*−308 (rs1800629) in the gene encoding tumour necrosis factor A and a deletion in intron 10 (rs9281523) in an adjacent gene encoding a spliceosome RNA helicase (*BAT1*). The minor alleles (denoted *2) mark carriage of the conserved 8.1 ancestral haplotype (AH), which has been associated with several immunopathologies, including type I diabetes [1, 26]. *BAT1 * (intron 10)*2 is a specific marker for the 8.1AH in Caucasians and in equivalent central MHC haplotypes found in Asians and Africans [29]. Ex vivo analyses suggest *TNF*−308*2 increases the transcription of TNF-α, but this may be a haplotypic effect [1] as it is not supported by in vitro analyses of reporter constructs [13]. TNF-α has potential to damage human brain cells and disrupt the physiology of neuronal cells [23] and is implicated in neuronal necroptosis in Alzheimers disease [37]. However meta-analyses report that associations between *TNF*−308 and age-associated dementia are restricted to East Asian populations, with protective effects seen amongst North European populations under a dominant model [17, 30]. Accordingly, carriage of the minor allele associated with a decreased prevalence of dementia in Danish centenarians [5]. In addition to their distinct *TNF* haplotypes [29], we find levels of CMV-reactive antibodies at least 100-fold higher in Indonesian HIV patients and healthy adults compared to equivalent Australian populations (unpublished data).

The strongest genetic cause of age-associated cognitive impairment is *APOE* ε4 [11]. Evidence supports involvement of pathways accelerating neurodegeneration, neurovascular dysfunction, neuroinflammation, oxidative stress, endosomal trafficking impairments, disruption of lipid and cellular metabolism and altered transcriptional regulation [28]. Such mechanisms have potential to impact on the replication of CMV or the ensuing pathology. We address the outcome in individuals studied over 50 years of age, drawn from a well-characterised cohort of older Caucasian Australians. It is likely that most or all had been exposed to CMV in the course of a normal life in the community, since the virus is transmitted by saliva and commonly acquired in childhood. We therefore segregate by levels of antibodies reactive with a lysate of CMV-infected fibroblasts quantitated over extensive serial dilutions. We propose that age and genotype alter associations between levels of CMV-reactive antibodies and neurocognitive functions.

## Materials and methods

### Study cohort

The Australian Imaging, Biomarkers and Lifestyle (AIBL) study is a nationwide project designed to discover factors that determine the development of Alzheimer’s disease. Participants of the AIBL study were evaluated, diagnosed and tested by neuropsychologists using internationally recognised tests [7]. The study cohort (*n* = 419) comprised individuals across a range of clinical classifications, including cognitively normal (CN; *n* = 236), mild cognitive impairment (MCI; *n* = 39), and Alzheimer’s disease (AD; *n* = 144). Cognitive assessments allowed the derivation of domain specific composite scores [6] and participants were assessed for brain amyloid-β (Aβ) burden using positron emission tomography [19]. Participants were classified as having either low (Aβ *low*) or high (Aβ *high*) brain Aβ burdens based on a centiloid (CL) threshold of 25 CL [14]. *APOE* (rs7412 and rs429358), *TNF*−308 (rs1800629) and *BAT1 (*intron 10, rs9281523) genotype data were derived from either direct genotyping of the specific single-nucleotide polymorphisms (SNP) using TaqMan genotyping assays, or from genome-wide SNP arrays performed across the AIBL cohort [9]. The study was approved by the Ethics Committees of Austin Health, St Vincent’s Health, Hollywood Private Hospital and Edith Cowan University. All participants gave written and informed consent.

### CMV–reactive antibodies and CMV DNA

Plasma antibodies were quantitated using CMV lysate prepared from human foreskin fibroblasts infected with CMV AD169 using an in-house indirect ELISA [25]. Plasma samples from CMV seropositive healthy individuals were used as a standard [allocated a value of 1000 arbitrary units (AU/mL)] and independently for quality control. Serial dilutions of each sample were used to obtain a value in AU/mL. Based on these measurements, patients were dichotomized into two groups: CMV seronegative and CMV seropositive. The cut-off for seropositivity (17255 AU/mL) was determined as three standard deviations above the mean of eight samples classified as seronegative using the ARCHITECT CMV IgG assay (Abbott Diagnostic Systems, Lake Forrest, IL). A nested PCR protocol based on primers targeted a 519 bp polymorphic region of *UL55* (glycoprotein B) [2, 32] was applied to detect active CMV replication in blood cells from a subset of participants (*n* = 200).

### Statistical analysis

Correlation analyses were carried out to explore the influence and direction of the relationship between CMV-reactive antibody levels and cognitive measures. Linear regression analyses were then employed to further investigate the associations between CMV levels and cognition while accounting for the potential effect of covariates. The regression models included age (years), sex (M/F), the presence of the *APOE* ε4 allele (binary, presence/absence) and education levels as covariates. The final model was constructed using a stepwise forward selection to achieve the best model fit based on the Akaike information criterion. In addition, a CMV x *TNF* genotype interaction term was included to assess the combined effect of these two variables on cognition. Cognition measures were modelled as the continuous outcome variables. Regression results were inspected to ensure that all the model assumptions were met including checks for residual normality, data linearity, independence and homoscedasticity. To account for regression model stability and robustness of findings, we used bootstrap resampling with 1000 repeats for each model. P values were adjusted using False discovery rate as introduced by Benjamini and Hochberg [4]. All statistical analyses were performed in R (http://www.R-project.org/).

## Results

### In bivariate analyses, only *APOE* genotype associated with cognitive scores

**Fig. 1:**
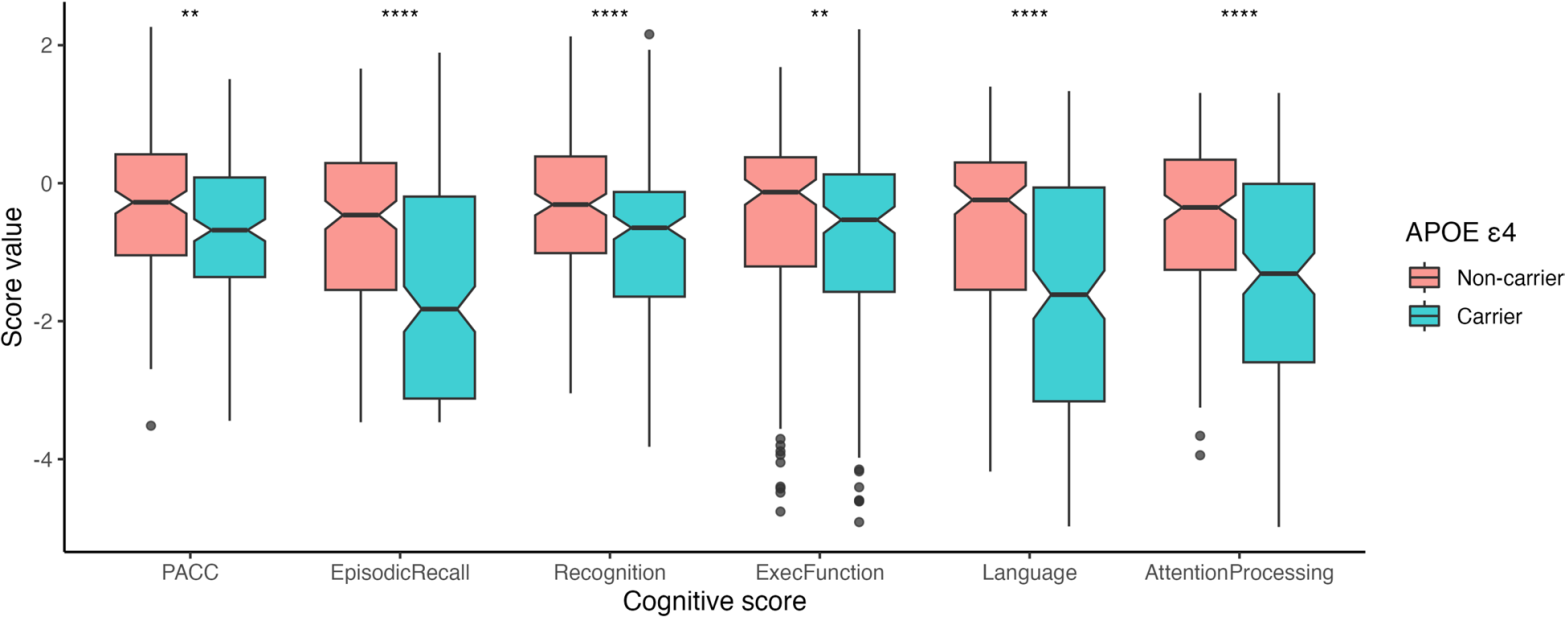
Effect of APOE ε4 allele on cognitive measures. Means were compared using t-tests, with the following symbols indicating statistical significance: ** *p* ≤ 0.01; *** *p* ≤ 0.001; **** *p* ≤ 0.0001

The presence of levels of CMV-reactive antibody > 3 standard deviations above the cut-off used in a commercial assay (defined here as seropositive) did not distinguish participants (*n* = 419) by their age, sex, brain Aβ burden, education, genotype or cognitive scores (Table 1). Similarly, the distribution of clinical classifications was balanced between CMV seropositive and seronegative participants (Table 1). Accordingly, CMV antibody levels did not correlate with age (*r* = 0.058, *p* = 0.23). Only one participant of 200 tested had CMV DNA in whole blood screened by nested PCR (data not shown), so active CMV replication was not assessed further. We confirmed that carriers of at least one copy of the *APOE* ε4 allele exhibited a significantly poorer performance over all cognitive domains (Fig. 1).

**Table 1:** Population demographics are not distinguished by levels of CMV–reactive antibodies

	Whole cohort	CMV seronegative	CMV seropositive	p-value
	(N=419)	(N=161)	(N=258)	
Sex (F/M)	213 / 206	74 / 87	139 / 119	0.132
Age (years)	71.1 [51.5, 89.1]	70.2 [53.2, 85.9]	71.4 [51.5, 89.1]	0.567
Cognitive Scores				
PACC	-0.79 [-5.42, 1.40]	-0.80 [-5.42, 1.35]	-0.75 [-4.51, 1.40]	0.822
Episodic Recall	-0.85 [-3.46, 1.89]	-1.05 [-3.46, 1.66]	-0.77 [-3.46, 1.89]	0.785
Recognition	-0.63 [-7.35, 1.31]	-0.76 [-4.42, 1.31]	-0.60 [-7.35, 1.31]	0.726
Exec Function	-0.50 [-3.82, 2.16]	-0.48 [-3.82, 2.16]	-0.50 [-3.57, 1.93]	0.855
Language	-0.41 [-6.45, 2.23]	-0.29 [-6.44, 1.68]	-0.48 [-6.45, 2.23]	0.38
Atten Processing	-0.43 [-3.52, 2.27]	-0.45 [-3.52, 1.57]	-0.43 [-2.77, 2.27]	0.564
Education				
0-6	4 (1.0%)	1 (0.6%)	3 (1.2%)	0.781
7-8	25 (6.0%)	11 (6.8%)	14 (5.4%)	
9-12	170 (40.6%)	60 (37.3%)	110 (42.6%)	
13-15	76 (18.1%)	28 (17.4%)	48 (18.6%)	
15+	124 (29.6%)	51 (31.7%)	73 (28.3%)	
Amyloid status				
Ab-Low	125 (29.8%)	54 (33.5%)	71 (27.5%)	0.33
Ab-High	215 (51.3%)	80 (49.7%)	135 (52.3%)	
APOEe4				
Absent	204 (48.7%)	81 (50.3%)	123 (47.7%)	0.688
Present	214 (51.1%)	80 (49.7%)	134 (51.9%)	
TNF-308				
TNF-308*1	274 (65.4%)	107 (66.5%)	167 (64.7%)	0.752
TNF-308*2	145 (34.6%)	54 (33.5%)	91 (35.3%)	
Diagnosis				
CN	236 (56.3%)	90 (55.9%)	146 (56.6%)	0.504
MCI	39 (9.3%)	12 (7.5%)	27 (10.5%)	
AD	144 (34.4%)	59 (36.6%)	85 (32.9%)

### CMV antibodies associated with protection but the effect was dependent on host genotype

In the subset of individuals classified as seropositive, brain Aβ burden (assessed using CL values) was negatively correlated with the levels of CMV-reactive antibodies (*r* = −0.153, *p* = 0.026, *n* = 206), consistent with a protective function for the antibodies. We sought to confirm this through correlations between levels of CMV-reactive antibodies and cognitive scores. Positive correlations were observed across three cognitive scores (PACC, Episodic Recall and Recognition) but only in individuals carrying *TNF*−308*2. Within this subpopulation, the correlations were stronger in individuals lacking the *APOE* ε4 allele (Table 2).

**Table 2:** Levels of CMV-reactive antibody and cognitive scores correlate directly in individuals who carry *TNFA*-308*2, notably without *APOE* ε4

	**Whole cohort**		TNFA-308*2		TNFA-308*2 without APOE ε4	
	*(N=419)*		*(N=145)*		*(N=73)*	
	r	*p.adj*	r	*p.adj*	r	*p.adj*
PACC	0.06	0.27	0.17	**0.04**	0.31	**0.01**
Episodic Recall	0.07	0.17	0.2	**0.02**	0.3	**0.01**
Recognition	0.07	0.18	0.22	**0.01**	0.3	**0.01**
Exec Function	0.06	0.23	0.13	0.13	0.21	0.08
Language	0.03	0.55	0.13	0.12	0.21	0.08
Atten Processing	-0.01	0.81	-0.01	0.94	0.07	0.55

**Fig. 2:**
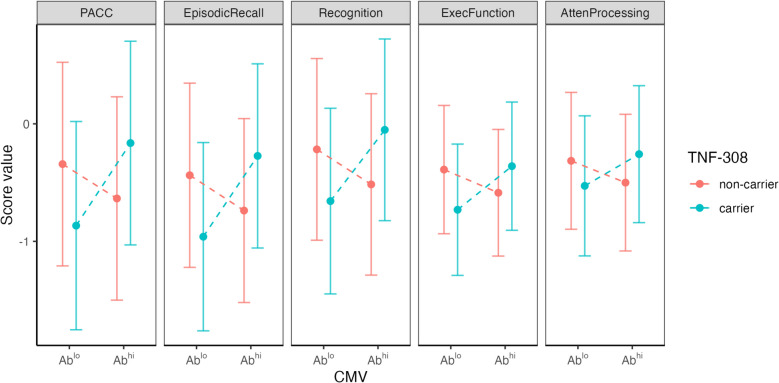
Interaction plot showing the combined effects of *TNF*-308*2 carriage and seropositivity on cross-sectional cognitive measures. Interaction effects were extracted from linear regressions. Models with significant interactions are displayed

Interactions between the effects of *TNF*−308 genotype and CMV were explored further in multivariable analyses adjusting for gender, age, education level and *APOE* genotype. Linear regressions revealed no significant effect of *TNF* genotype per se*,* but displayed significant interactions between *TNF*−308*2 genotype and CMV antibodies (Table 3A, Fig. 2).

**Table 3:** The effects of CMV antibodies interacted with those of genotype but not ε4

A) Whole Cohort(N=419)	Overall model		CMV x TNF-308*2 interaction			
	r2	p.model	estimate	SE [95% CI]	p	p.adj
PACC	0.221	2.50E-15	0.985	0.32 [0.35, 1.62]	0.002	0.005
Episodic Recall	0.248	6.10E-18	0.973	0.29 [0.4, 1.54]	8.60E-04	0.005
Recognition	0.199	6.50E-13	0.893	0.29 [0.33, 1.46]	0.002	0.005
Exec Function	0.174	1.50E-11	0.574	0.23 [0.13, 1.02]	0.001	0.017
Language	0.155	2.90E-09	0.506	0.32 [-0.13, 1.14]	0.12	0.12
Atten Processing	0.179	8.30E-12	0.471	0.21 [0.05, 0.89]	0.003	0.034
B) Whole Cohort(N=419)	Overall model		CMV x APOE e4 interaction			
	r2	p.model	estimate	SE [95% CI]	p	p.adj
PACC	0.2	6.40E-14	-0.027	0.19 [-0.4, 0.34]	0.89	0.89
Episodic Recall	0.224	3.50E-16	0.063	0.19 [-0.31, 0.43]	0.74	0.89
Recognition	0.175	2.20E-11	-0.065	0.19 [-0.44, 0.31]	0.74	0.89
Exec Function	0.161	7.30E-11	0.142	0.19 [-0.24, 0.52]	0.46	0.89
Language	0.148	4.00E-09	-0.065	0.2 [-0.46, 0.33]	0.74	0.89
Atten Processing	0.167	2.90E-11	0.094	0.19 [-0.29, 0.48]	0.63	0.89

We were able to repeat the analyses using the *BAT1 *(intron 10) allele genotyped in 200 samples. Amongst these individuals, 36.5% carried *TNF*−308*2 and 28.9% carried *BAT1 *(intron 10)*2—consistent with *BAT1 *(intron 10) being a more specific marker of the 8.1AH [29]. PACC scores and CMV-reactive antibodies correlated directly in carriers of *TNF*−308*2 (*r* = 0.23, p.adj = 0.09) and *BAT1 *(intron 10)*2 (*r* = 0.28, p.adj = 0.06), contrasting with *r* < 0.08 in individuals without the minor allele. This pattern was repeated with episodic recall, recognition and executive function (data not shown), suggesting that the effects of *TNF*−308*2 align with the 8.1AH.

We also sought interaction terms between *APOE* genotype and CMV. None were significant in the entire cohort (Table 3B) or in individuals stratified by age (data not shown).

### Associations between CMV and *TNF*-308 genotype were clearer in younger participants.

The age of participants varies between published studies and so could explain diverse associations between CMV and neurocognition. We were able to address this because participants sampled here were aged 56 to 89 years. In bivariate analyses, age correlated inversely with most cognitive domains but not with levels of CMV-reactive antibody (data not shown). To explore this further, we repeated the analyses after dichotomising the samples around the median age (71.1 years) (Table 4). In the younger group, interaction terms describing PACC scores, executive function and recognition were significant and marginal associations were seen with 3 other domains. This was not evident amongst the older participants.

**Table 4:** Associations between CMV antibodies and *TNF*-308 genotype were only significant in younger participants

Age < 71.1 yrs (N=209)	Overall model		C MV x TNF-308 interaction			
	r2	p.model	estimate	SE [95% CI]	p	p.adj
PACC score	0.266	8.70E-09	1.274	0.39 [0.5, 2.05]	1.40E-03	0.004
Episodic Recall	0.292	3.50E-10	1.269	0.35 [0.57, 1.97]	4.20E-04	0.002
Recognition	0.266	6.60E-09	1.003	0.33 [0.35, 1.66]	3.00E-03	0.006
Exec Function	0.297	5.80E-11	0.579	0.28 [0.03, 1.13]	3.90E-02	0.058
Language	0.154	6.70E-04	0.261	0.35 [-0.42, 0.94]	4.50E-01	0.45
Atten Processing	0.182	2.30E-05	0.405	0.3 [-0.18, 0.99]	1.80E-01	0.216
Age > 71.1 yrs (N=210)	Overall model		CMV x TNF-308 interaction			
	r2	p.model	estimate	SE [95% CI]	p	p.adj
PACC score	0.273	6.60E-09	0.72	0.46 [-0.19, 1.63]	1.20E-01	0.22
Episodic Recall	0.305	2.50E-10	0.554	0.43 [-0.29, 1.4]	2.00E-01	0.22
Recognition	0.244	5.40E-07	0.678	0.47 [-0.25, 1.6]	1.50E-01	0.22
Exec Function	0.143	1.50E-03	0.419	0.34 [-0.26, 1.1]	2.20E-01	0.22
Language	0.18	7.70E-05	0.659	0.53 [-0.39, 1.71]	2.20E-01	0.22
Atten Processing	0.164	2.30E-04	0.427	0.3 [-0.17, 1.02]	1.60E-01	0.22

### Associations between CMV and amyloid plaque are *APOE *ε4-dependent and occur in younger participants

Centiloid (CL) values provide a continuous measure of amyloid plaque accumulation. Among CMV seropositive individuals, CL values were significantly correlated with CMV-reactive antibody levels (*r* = –0.153, *p* = 0.026, *n* = 206). However, this relationship was not observed when examining the full cohort (*r* = –0.019, *p* = 0.360, *n* = 340). To identify sub-groups where CMV may impact upon amyloid, the cohort was stratified by age and genotype. The correlation between CL and CMV antibodies was not strengthened by stratification based on *TNF*−308 (*TNF*−308*2 carriers, *r* = −0.09, *p* = 0.320, *n* = 118). However, it was restricted to individuals lacking the *APOE* ε4 allele (*r* = −0.329, *p* = 0.012, *n* = 57; *cf* with *APOE* ε4 *r* = 0.052, *p* = 0.478, *n* = 186). This pattern was also observed in the younger subpopulation (individuals under 71.1 years of age) (without *APOE* ε4, *r* = −0.224, *p* = 0.042, *n* = 82), whereas no association was evident in the older group (without *APOE* ε4, *r* = −0.113, *p* = 0.561, *n* = 71). Overall, we conclude that CMV may contribute to plaque accumulation during earlier stages of aging (before age 71.1), particularly in individuals not genetically predisposed to accelerated pathology by carriage of *APOE ε4*.

## Discussion

The data suggest that *TNF*−308*2 genotype and levels of CMV antibodies may act synergistically to protect cognitive function in younger participants (under 71.1 years of age), particularly among those without the *APOE ε4* allele. However, neither CMV nor *TNF*−308*2 had a significant effect alone. Inverse associations between CMV antibodies and cognitive impairment appear counterintuitive as it is generally assumed that antibody levels are proportional to the persistent burden of CMV. Use of antibodies to assess the burden of CMV associated with age-associated disorders is common in the literature, including our study of a West Australian population [Busselton cohort; 56(40–80) years old] where CMV antibody levels associated with all-cause mortality [16]. However, extrapolation of a direct link between antibody levels and viral burden to the present data would imply that CMV itself protects cognitive function. As we are aware of no evidence to support this notion, we suggest that antibody controls CMV replication in extreme old age, so *higher* levels antibody associate with a *lower* burden of CMV. A protective role for antibody is plausible as memory B-cell responses are maintained into old age, whilst responses to novel antigens decline [36]. Since most CMV infections are initiated early in life, the antibody detected by ELISA may be produced by senescent B-cells maintaining a high secretory profile with little proliferative capacity [10]. Accordingly older individuals rarely experience acute CMV infections (as seen in transplant recipients), so protective responses sufficient to control the replication of CMV are probably maintained in old age [36]. Here only one participant of the 200 tested was CMV DNA positive when assessed using a nested PCR applied to whole blood, so an effect of CMV is presumably not predicated on viral replication proceeding at a level that could be detected in whole blood. This is plausible because CMV is known to persist in “sleepless latency” [24] with ongoing transcription of several immune-related genes. Examples include homologues of human chemokine receptors (US28) and interleukin-10 (cmvIL-10). Genes encoding these proteins vary between clinical isolates, providing a mechanism for individual variation in the rate of cognitive loss [32, 34, 35].

The direct relationship between cognitive performance and CMV antibodies was greatest in individuals with carrying *TNF*−308*2 and not the *APOE* ε4 allele. This was supported by multivariate analyses showing significant interaction terms between the *TNF* genotype and CMV antibodies. Considering the *TNF* genotype alone, we noted that meta-analyses have implicated *TNF*−308*2 as a risk factor for AD in Eastern Asians, though not in Europeans [17, 30]. This link with ethnicity may reflect haplotypic associations as *TNF*−308*2 is restricted to a haplotype marked by *BAT1 *(intron 10)*2 in Asians, but is also carried within a distinct haplotype without this allele in Caucasians [29]. However, carriage of the minor allele was associated with protection in Danish centenarians but not octogenarians [5], providing a precedent for the protection seen here. Unfortunately their study did not assess CMV.

Brain Aβ burden was used to seek preliminary evidence regarding the mechanisms targeted by CMV. Antibody levels correlated inversely with brain Aβ burden, suggesting a possible role for CMV in the formation or deposition of Aβ. This was restricted to younger individuals lacking the *APOE* ε4 allele and was not systematically affected by the *TNF*−308 genotype. In accordance with a protective role, levels of CMV-reactive antibodies correlated directly with cognitive performance. This was restricted to individuals carrying *TNF*−308*2, notably those not carrying the *APOE* ε4 allele. Accordingly, regression analyses adjusted for the *APOE* ε4 genotype identified significant interactions between *TNF* genotype and CMV antibody levels in the whole cohort and in the youner subpopulation. Taken together these findings lead us to hypothesise that CMV promotes formation or deposition of Aβ in the absence of the *APOE* ε4 allele and may drive a later step of neuropathological process between Aβ and cognitive loss. This was clearest in individuals with *TNF*−308*2. One would expect such people to have a higher inflammatory response to CMV so perhaps it is critical that they have antibodies to control replication of the virus.

The limitations of our study include the relatively small sample size and the cross-sectional design. However, we identified several factors that should be considered in future studies of the role of CMV in cognitive impairment. We suggest that CMV antibodies are able to control viral replication in old age and so may play a protective role in carriers of the minor allele of *TNF*−308. This was significant in younger individuals and could be eclipsed by advanced age or carriage of the *APOE* ε4 allele. Our data do not distinguish whether CMV creates an environment that enhances the neuropathic effects of TNF (which is plausible but has never been addressed) from a scenario where TNF promotes the reactivation of latent CMV, as shown in several in vitro studies (eg: [8]). However in either case, the interactions described may explain disagreements in the literature regarding the effects of CMV and *TNF*−308.

## Data Availability

The data supporting the findings of this study are available in the following repository: 10.25919/g0hs-t095.

## Abbreviations

AD: Alzheimer's Disease
AIBL: Australian Imaging Biomarkers and Lifestyle
APOE: Apolipoprotein E
CL: Centiloid
CMV: Human Cytomegalovirus
MHC: Major Histocompatibility Complex
MCI: Mild Cognitive Impairment
PACC: Preclinical Cognitive Composite Score
TNF: Tumor Necrosis Factor
